# Trends in the perceived body size of adolescent males and females in Scotland, 1990–2014: changing associations with mental well-being

**DOI:** 10.1007/s00038-017-0997-y

**Published:** 2017-07-01

**Authors:** Ross D. Whitehead, Alina Cosma, Jo Cecil, Candace Currie, Dorothy Currie, Fergus Neville, Jo Inchley

**Affiliations:** School of Medicine, University of St Andrews, St Andrews, Fife, Scotland UK

**Keywords:** Body image, Body size perception, Overweight, Underweight, Adolescents, Mental well-being

## Abstract

**Objectives:**

This paper explores trends in Scottish adolescents’ body size perceptions and associated mental well-being outcomes.

**Methods:**

Data were collected on Scottish 11-, 13-, and 15-year-olds by the Health Behaviour in School-aged Children study between 1990 and 2014 (*n* = 42,312). Logistic regression was used to examine changes in the prevalence of over- and underweight perceptions. Ordinal and linear regressions were used to examine changes in the association between body perception and mental well-being.

**Results:**

Little change was observed in over- or underweight perceptions. However, relative to those perceiving their body as ‘about right’, those perceiving themselves as overweight reported decreasing confidence (all groups), decreasing happiness (11- and 13-year-old girls), and increasing psychological health symptoms (all girls and 15-year-old boys). Perceived underweight is associated with poor well-being, especially in males, but we present little evidence that this is a recent phenomenon.

**Conclusions:**

We present evidence suggesting that the association between body size perception and poor mental health in adolescence is changing over time. This may play a role in the recently observed worsening of mental well-being in Scottish adolescents.

**Electronic supplementary material:**

The online version of this article (doi:10.1007/s00038-017-0997-y) contains supplementary material, which is available to authorized users.

## Introduction

Conspicuous physical development during adolescence draws attention to one’s body shape and size, instigating comparison against peers and subjective ideals (Voelker et al. 2015). This can lead to dissatisfaction with one’s body, where self-perception is discordant with a desired target (Cash and Szymanski 1995). For decades, the desirable body shape for females has been thin (Sypeck et al. 2004), with considerable pressure to conform to this target, even in preadolescence (Blowers et al. 2003). Consequently, overweight perceptions are common amongst adolescent girls in Europe and North America. Despite approximately 10% of 15-year-old females being classified as overweight or obese, 40% of this group perceive that they are overweight (Inchley et al. 2016). Around one in five 15-year-old males are classified as overweight or obese, which is concordant with the proportion of male adolescents perceiving that they are overweight (Inchley et al. 2016). Both overweight and underweight perceptions are common among boys, with around 20% of non-overweight boys perceiving that they are underweight (Quick et al. 2014).

Perceiving one’s body as overweight is associated with poor mental well-being outcomes in adolescence including low self-esteem (Williams and Currie 2000), depression (Atlantis and Ball 2008), and anxiety (Cash and Fleming 2002). This perception can also influence physical health through maladaptive weight manipulation strategies such as binge eating, smoking, and purging (Neumark-Sztainer et al. 2006). Perceived overweight and its consequences are far from transient as their establishment in adolescence is likely to track into adulthood (Blashill and Wilhelm 2014; Friestad and Rise 2004). Longitudinal evidence indicates that perceiving oneself as overweight is associated with later weight gain, even for those with normal baseline weight (Robinson et al. 2015).

Monitoring of adolescent weight perception and associated comorbidities is essential for evaluation and improvement of current and future public health, especially given worldwide patterns in the prevalence of obesity (World Health Organization 2015). It is reasonable to expect that the increasing prevalence of overweight and obesity might reduce population-level overweight perceptions as individuals compare themselves against increasingly heavier norms (Strahan et al. 2006). Indeed, a recent study across 24 European and North American countries found that overweight adolescents became less likely to perceive themselves as overweight between 2002 and 2010, despite an increase in country-level self-reported BMI over this period (Quick et al. 2014). However, the obesity epidemic has been accompanied by growing discourse around weight-related issues, particularly on the role of individual responsibility (Brownell et al. 2010; Taylor 2011). As such, a rise in the discussion of weight-related issues among adolescents may increase the potential for body dissatisfaction and associated negative consequences, in part via increased stigmatization (O’Dea 2005).

The proportion of Scottish adolescents perceiving their body as overweight is amongst the highest in Europe and North America, despite their weight being close to the average of these regions (Inchley et al. 2016). There is, therefore, a strong incentive to monitor the self-perceptions of young people in Scotland to inform preventive strategies against body image dissatisfaction and its comorbidities. This paper examines 24 years (1990–2014) of cross-sectional survey data on perceived body size from the Health Behaviour in School-aged Children (HBSC) study in Scotland among 11-, 13-, and 15-year-olds. Age differences in body size perception will be examined within this sensitive developmental window. In addition, with data on mental well-being indicators available between 1994 and 2014, we explore whether the association between mental well-being and perceived body size has changed over time. Specifically, by comparing trends across different weight-perception groups, we examine whether any change in this association contributes to the recently observed worsening of Scottish adolescents’ happiness, confidence, and psychological health symptoms (Currie et al. 2015).

## Methods

Existing data are used from seven rounds of the Scottish HBSC study. This cross-sectional study has been conducted quadrennially since 1990, with the latest data collection in 2014. For each survey, a nationally representative sample of pupils in three school grades (P7, S2, and S4; average ages approximately 11.5, 13.5, and 15.5, hereafter 11, 13, and 15 years, respectively) completed a paper-based questionnaire in a classroom setting. An average of 6125 (SD ±2287.5) pupils participated per survey, with 42,873 pupils participating since 1990. Over the period 1990–2014, class response rates were between 69 and 86%. Information on pupil response rates has been collected routinely since 2002. Over this period, pupil response rates have ranged between 83 and 93%.

Scotland follows the international HBSC protocol (Currie et al. 2014). Survey methods for the Scottish survey are described in detail elsewhere (Currie et al. 2015). Briefly, the sample for each survey round was stratified proportionately by school grade, local authority, and school type (independent versus state funded). Pupils were selected via cluster sampling of school classes. No inclusion criteria were applied beyond the requirement to be within a randomly selected school class. Pupils not reporting body size perception were excluded from analyses (1.3%).

### Compliance with ethical standards

For survey rounds between 1990 and 2010, ethical approval was granted by the Moray House School of Education Ethics Committee, University of Edinburgh. The 2014 survey was approved by the University of St Andrews Teaching and Research Ethics Committee. Prior informed consent was obtained at local authority, school, parent, and pupil levels.

### Perceived body size

For all seven survey cycles, pupils were asked to report whether they perceived their body to be “Much too thin”, “A bit too thin”, “About the right size”, “A bit too fat”, or “Much too fat”. The former two options were considered here as ‘perceived underweight’ with the latter two as ‘perceived overweight’. Between 1990 and 1998, participants could also select “I don’t think about it”. For the purposes of analysis, “I don’t think about it” and “About the right size” are assumed to indicate the absence of perceived over- or underweight (henceforth ‘about right’).

### Body mass index (BMI)

BMI (kg/m^2^) was calculated from self-reported weight and height, available from a total of 8160 pupils from 2006 onwards.

### Well-being outcomes

#### Confidence

From 1994 onwards, pupils were asked how often they feel confident in themselves with five response options (“Never”, “Hardly ever”, “Sometimes”, “Often”, and “Always”).

#### Happiness

From 1994 onwards, pupils were asked “In general, how do you feel about your life at present?”, with four response options (“I’m not happy at all”, “I don’t feel very happy”, “I feel quite happy”, and “I feel very happy”).

#### Psychological symptoms

From 1994 onwards, pupils reported the frequency of eight health symptoms in the past 6 months (nervousness, bad temper, feeling low, sleep difficulties, headaches, stomach aches backaches, and dizziness), with five response options per symptom (“About every day”, “More than once a week”, “About every week”, “About every month”, and “Rarely or never”). Responses were combined via principal components analysis. Mirroring international validation of this scale (Haugland et al. 2001), the first factor of a rotated two-factor solution explained 43.1% of the variation in health symptom frequency. This first factor reflects psychological health symptom frequency, with the strongest loadings for nervousness (*λ* = 0.76), bad temper (*λ* = 0.72), feeling low (*λ* = 0.71), and sleep difficulties (*λ* = 0.63). The remaining health symptoms correlated less strongly with this factor (all *λ* < 0.34). In the present analysis, we focus on the first factor representing psychological symptoms as these are more pervasive than somatic symptoms in Scotland, and have shown more change in recent years (Currie et al. 2011, 2015). Higher factor scores reflect increased psychological health symptom burden.

### Statistical analyses

All analyses are stratified by gender as the nature of body weight perception differs between boys and girls (Inchley et al. 2016; Voelker et al. 2015). The SPSS (v.22) complex samples toolkit was used to adjust for survey design (clustering of pupils within local authority and school each within survey year). For all regression analyses, data set weights were applied as appropriate per survey to achieve national representativeness with respect to local authority area, school denomination, gender, and school urban–rural classification. Where age categories are combined, additional weighting is applied to ensure equal representation. Survey year is treated as a linear continuous variable throughout.

To examine age differences in adolescents’ weight perceptions, a series of binary logistic regressions were conducted combining data across all survey years (see Supplementary Tables S1 and S2, models 1a and 1b). Separate analyses were conducted with either over- or underweight perception as the binary-dependent variable. These binary variables coded either over- or underweight perception against all other responses. The sole independent variable, age category, was dummy coded as 11, 13, and 15 years, with one category omitted to act as reference, depending on the pairs of age categories being compared. The overall (all age categories combined) effect of survey year on over- and underweight perceptions was tested using separate binary logistic regressions, with survey year (treated as continuous) as the only independent variable (see Supplementary Tables S1 and S2, model 2). To test for change over time in any association between age and over- or underweight perception, further logistic regressions were conducted, allowing for interactions between age and survey year (see Supplementary Tables S1 and S2, models 3a and 3b). Where a significant interaction was observed, model 2 was repeated per age category to further elucidate any effect of survey year.

Associations between body size perception and categorical mental well-being outcomes (confidence and happiness) were tested in a series of ordinal regressions, treating these outcomes as ordinal-dependent variables. For these analyses, the main effects of body size perception (dummy coded as perceived underweight, perceived overweight or ‘about right’, with the latter as reference category) and survey year (centred on 1994) were entered as independent variables. To examine whether any association between well-being outcomes and perceived body size has changed over time, interaction terms between over- and underweight perception and survey year were also entered. The associations between body size perception and psychological symptoms (a continuous variable) were tested using similar structured general linear models. Parameter estimates for all models are given in Table 3.

## Results

The main analysis set is formed of 42,312 pupils reporting body size perception since 1990. The numbers available for each well-being outcome are slightly lower than this due to missing responses (see Tables 1 and 2 for descriptive statistics).

**Table 1:** Descriptive (unweighted) statistics of main sample, Scotland 1990–2014

	Boys	Girls	Total
	*N* (%)	*N* (%)	*N* (%)
School grade			
Primary 7 (11-year-old)	7489 (35.8)	7408 (34.6)	14,897 (35.2)
Secondary 2 (13-year-old)	6984 (33.4)	7183 (33.5)	14,167 (33.5)
Secondary 4 (15-year-old)	6424 (30.7)	6824 (31.9)	13,248 (31.3)
Total	20,897 (100)	21,415 (100)	42,312 (100)
Survey year			
1990	1899 (9.1)	2119 (9.9)	4018 (9.5)
1994	2378 (11.4)	2525 (11.8)	4903 (11.6)
1998	2724 (13.0)	2826 (13.2)	5550 (13.1)
2002	2238 (10.7)	2153 (10.1)	4391 (10.4)
2006	3008 (14.4)	3082 (14.4)	6090 (14.4)
2010	3288 (15.7)	3402 (15.9)	6690 (15.8)
2014	5362 (25.7)	5308 (24.8)	10,670 (25.2)
Total	20,897 (100)	21,415 (100)	42,312 (100)
Body size perception^a^			
Perceived underweight	3308 (15.8)	2138 (10.0)	5446 (12.9)
‘About right’	12,556 (60.1)	10,174 (47.5)	22,730 (53.7)
Perceived overweight	5033 (24.1)	9103 (42.5)	14,136 (33.4)
Total	20,897 (100)	21,415 (100)	42,312 (100)
Confidence			
Never	646 (3.4)	1137 (5.9)	1783 (4.7)
Hardly ever	152 (6.6)	2799 (14.6)	4051 (10.7)
Sometimes	4432 (23.5)	6586 (34.3)	11,018 (29.0)
Often	8114 (43.1)	6301 (32.8)	14, 415 (37.9)
Always	4390 (23.3)	2372 (12.4)	6762 (17.8)
Total	18,834 (100)	19,195 (100)	38,029 (100)
Happiness			
I’m not happy at all	263 (1.4)	432 (2.3)	695 (1.8)
I don’t feel very happy	1115 (5.9)	1973 (10.3)	3088 (8.1)
I feel quite happy	8410 (44.6)	9480 (49.4)	17,890 (47.0)
I feel very happy	9079 (48.1)	7301 (38.1)	16,380 (43.0)
Total	18,867 (100)	19,186 (100)	38,053 (100)
Psychological health symptoms^b^	18,410	18,929	37,339

^a^Between 1990 and 1998, participants could also select that “I don’t think about it”. For the purposes of analysis, this option is coded as ‘about right’. Those responding that they think their body is “a bit too thin” or “much too thin” are categorised ‘perceived underweight’, and those responding that their body is “a bit too fat” or “much too fat” are categorised ‘perceived overweight’

^b^Principal component analysis was used to generate an index of the following psychological symptoms: nervousness, bad temper, feeling low, and sleep difficulties

**Table 2:** Descriptive (unweighted) statistics of perceived body size by gender, age, and survey year, Scotland 1990–2014

		Boys			Girls		
		Perceived underweight *N* (%)	‘About right’ *N* (%)	Perceived overweight *N* (%)	Perceived underweight *N* (%)	‘About right’ *N* (%)	Perceived overweight *N* (%)
1990							
11-year-old		76 (11.9)	442 (69.3)	120 (18.8)	71 (10.3)	385 (56.0)	231 (33.6)
13-year-old		99 (16.0)	379 (61.2)	141 (22.8)	75 (11.1)	323 (47.9)	277 (41.0)
15-year-old		137 (21.3)	374 (58.3)	131 (20.4)	70 (9.2)	298 (39.4)	389 (51.4)
1994							
11-year-old		117 (12.0)	663 (68.0)	195 (20.0)	120 (12.0)	555 (55.4)	327 (32.6)
13-year-old		122 (15.9)	449 (58.5)	196 (25.6)	91 (11.5)	369 (46.5)	334 (42.1)
15-year-old		131 (20.6)	365 (57.4)	140 (22.0)	57 (7.8)	266 (36.5)	406 (55.7)
1998							
11-year-old		144 (13.6)	681 (64.4)	233 (22.0)	121 (11.9)	535 (52.7)	359 (35.4)
13-year-old		133 (15.3)	494 (56.7)	245 (28.1)	102 (11.3)	352 (39.0)	449 (49.7)
15-year-old		157 (19.8)	439 (55.3)	198 (24.9)	81 (8.9)	308 (33.9)	519 (57.2)
2002							
11-year-old		128 (13.8)	593 (63.8)	209 (22.5)	99 (12.3)	434 (53.8)	274 (34.0)
13-year-old		112 (15.3)	425 (58.2)	193 (26.4)	96 (12.4)	324 (41.8)	356 (45.9)
15-year-old		124 (21.5)	335 (58.0)	119 (20.6)	53 (9.3)	219 (38.4)	298 (52.3)
2006							
11-year-old		109 (13.4)	539 (66.1)	167 (20.5)	122 (13.8)	510 (57.8)	250 (28.3)
13-year-old		172 (15.6)	613 (55.6)	317 (28.8)	118 (10.5)	513 (45.8)	489 (43.7)
15-year-old		199 (18.2)	621 (56.9)	271 (24.8)	89 (8.2)	468 (43.3)	523 (48.4)
2010							
11-year-old		158 (15.5)	651 (63.8)	211 (20.7)	124 (11.9)	663 (63.9)	251 (24.2)
13-year-old		161 (15.2)	591 (55.9)	306 (28.9)	101 (9.7)	476 (45.8)	463 (44.5)
15-year-old		246 (20.3)	615 (50.8)	349 (28.8)	100 (7.6)	536 (40.5)	688 (52.0)
2014							
11-year-old		244 (11.9)	1422 (69.3)	387 (18.9)	169 (8.5)	1287 (65.1)	521 (26.4)
13-year-old		258 (14.1)	1089 (59.3)	489 (26.6)	166 (8.9)	826 (44.1)	883 (47.1)
15-year-old		281 (19.1)	776 (52.7)	416 (28.2)	113 (7.8)	527 (36.2)	816 (56.0)

Between 1990 and 1998, participants could also select that “I don’t think about it”. For the purposes of analysis, this option is coded as ‘about right’. Those responding that they think that their body is “a bit too thin” or “much too thin” are categorised ‘perceived underweight’, and those responding that their body is “a bit too fat” or “much too fat” are categorised ‘perceived overweight’

### Perceived underweight

Overall 10.0% of girls and 15.8% of boys perceived that they are underweight (Table 1) (see Table 2 for the prevalence of perceived underweight disaggregated by gender, age, and survey year and also see Supplementary Table S1 for further details on the models used to examine age differences and time trends in perceived underweight). The likelihood of perceived underweight was equivalent between 11- and 13-year-old girls [*χ* ^2^ (1) = 0.84, *p* = .361]. Fifteen-year-old girls exhibited lower levels of perceived underweight relative to both groups of younger girls (both *χ* ^2^ > 19.51, *p* < .001). Among girls, the prevalence of perceived underweight reduced slightly over time [*F* (1, 1617) = 9.86, *p* = .002], with no relative change between age groups (all interactions *F* < 0.08, *p* > .774)

Among boys, the likelihood of perceived underweight increased with age (all comparisons *χ* ^2^ > 10.08, *p* < .002). No effect of survey year was seen on perceived underweight among boys [*F* (1, 1617) = 1.052, *p* = .305] with no relative change in perceived underweight between age groups (all interactions *F* < 2.97, *p* > .085).

### Perceived overweight

Overall, 42.5% of girls and 24.1% of boys perceived themselves as overweight (Table 1) (see Table 2 for the prevalence of perceived overweight disaggregated by gender, age, and survey year and also see Supplementary Table S2 for further details on the models used to examine age differences and time trends in perceived overweight). The likelihood of perceived overweight increased with age among girls (all comparisons *χ* ^2^ > 75.2, *p* < .001). Since 1990, 11-year-old girls showed a linear decrease in perceived overweight [*F* (1, 1617) = 34.54, *p* < .001]. Overweight perceptions among 13- and 15-year-old girls did not change over this period (both *F* < 0.44, *p* > .51).

Among boys, the likelihood of perceived overweight was higher among 13- and 15-year-olds relative to 11-year-olds (both *χ* ^2^ > 35.54, *p* < .001). Fifteen-year-old boys exhibited lower levels of perceived overweight relative to 13-year-old boys *χ* ^2^ (1 = 4.50, *p* = .034), but whereas 11- and 13-year-old boys did not change over time (both *F* < 2.57, *p* > .109), 15-year-old boys showed a linear increase in perceived overweight between 1990 and 2014 [*F* (1, 1617) = 20.17, *p* < .001].

### Association between perceived body size and mental well-being outcomes

The following analyses are further stratified by age group as the analysis above indicates heterogeneity in perceived body size over time.

#### Confidence

**Fig. 1:**
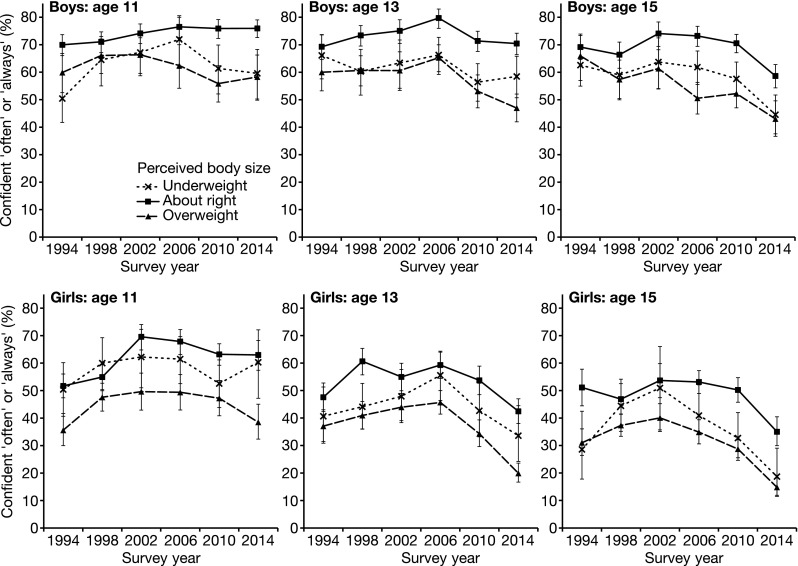
Relationship between Scottish adolescents’ confidence and perceived body size 1994–2014. The proportion of boys and girls aged 11, 13, and 15 reporting that they are confident “often” or “always” is presented for those perceiving their body is underweight (*dashed line*, *crosses*), overweight (*dashed line*, *triangles*), or ‘about right’(*solid line*, *squares*). For the 1994 and 1998 surveys, participants could also select that “I don’t think about it”. For the purposes of analysis, this option is coded as ‘about right’. *Error bars* represent ±95% CI. Scotland 1994–2014

The relationship between confidence and perceived body size over the period 1994–2014 is demonstrated in Fig. 1. This relationship is formally examined in Table 3, which presents the results of ordinal regressions testing main effects of body size perception and survey year, and their interactive effect on confidence for each age/gender permutation. For boys of all observed ages and 15-year-old girls, those perceiving themselves as underweight exhibited reduced confidence relative to those perceiving themselves as ‘about right’. Perceived overweight was associated with lower confidence for all age/gender permutations.

**Table 3:** Associations between perceived body size and mental well-being outcomes, Scotland 1994–2014

	Boys			Girls		
	11 years	13 years	15 years	11 years	13 years	15 years
Confidence^a^						
‘About right’^b^ (ref)						
Perceived underweight	0.65 (0.50, 0.85)**	0.66 (0.51, 0.87)**	0.69 (0.55, 0.86)***	1.03 (0.76, 1.39)	0.76 (0.57, 1.03)	0.68 (0.48, 0.98)*
Perceived overweight	0.71 (0.58, 0.86)***	0.75 (0.61, 0.93)**	0.74 (0.58, 0.93)**	0.62 (0.52, 0.73)***	0.63 (0.53, 0.75)***	0.58 (0.48, 0.70)***
Year^c^	1.01 (1.00, 1.02)	1.00 (0.99, 1.01)	0.99 (0.97, 1.00)^**^	1.02 (1.01, 1.03)**	0.98 (0.97, 0.99)***	0.97 (0.96, 0.98)***
Year^c^ × ‘about right’^b^ (ref)						
Year^c^ × perceived underweight	0.99 (0.97, 1.01)	0.99 (0.97, 1.02)	0.99 (0.97, 1.01)	0.98 (0.96, 1.00)	0.99 (0.97, 1.02)	0.99 (0.96, 1.01)
Year^c^ × perceived overweight	0.97 (0.96, 0.99)**	0.97 (0.95, 0.98)***	0.97 (0.95, 0.99)***	0.97 (0.96, 0.99)***	0.97 (0.95, 0.98)***	0.97 (0.96, 0.99)***
Happiness^a^						
‘About right’^b^ (ref)						
Perceived underweight	0.71 (0.52, 0.96)*	0.65 (0.49, 0.86)**	0.56 (0.42, 0.74)***	0.90 (0.68, 1.19)	0.77 (0.57, 1.04)	0.71 (0.47, 1.07)
Perceived overweight	0.43 (0.34, 0.55)***	0.45 (0.35, 0.58)***	0.51 (0.40, 0.65)***	0.40 (0.33, 0.48)***	0.40 (0.33, 0.48)***	0.42 (0.34, 0.52)***
Year^c^	1.02 (1.01, 1.03)***	1.01 (1.00, 1.02)^*^	1.00 (0.99, 1.01)	1.03 (1.02, 1.04)***	1.02 (1.01, 1.03)***	1.00 (0.99, 1.01)
Year^c^ × ‘about right’^b^ (ref)						
Year^c^ × perceived underweight	0.98 (0.96, 1.01)	0.99 (0.96, 1.01)	1.00 (0.98, 1.03)	0.97 (0.94, 0.99)**	0.99 (0.97, 1.02)	0.98 (0.95, 1.02)
Year^c^ × perceived overweight	0.99 (0.97, 1.02)	1.00 (0.98, 1.02)	0.99 (0.97, 1.01)	0.98 (0.96, 1.00)*	0.98 (0.96, 0.99)**	0.99 (0.97, 1.01)
Psychological symptoms^d^						
‘About right’^b^ (ref)						
Perceived underweight	0.26 (0.13, 0.39)***	0.03 (−0.12, 0.17)	0.28 (0.15, 0.40)***	0.17 (0.00, 0.33)*	0.22 (0.03, 0.40)*	0.26 (0.05, 0.46)*
Perceived overweight	0.26 (0.15, 0.38)***	0.20 (0.08, 0.33)***	0.14 (0.03, 0.26)*	0.29 (0.19, 0.39)***	0.23 (0.12, 0.33)***	0.21 (0.10, 0.33)***
Year^c^	−0.01 (−0.02, −0.01)***	−0.01 (−0.01, 0.00)^**^	0.01 (0.00, 0.01)**	−0.01 (−0.02, −0.01)***	0.00 (−0.01, 0.00)	0.01 (0.01, 0.02)***
Year^c^ × ‘about right’^b^ (ref)						
Year^c^ × perceived underweight	0.00 (−0.01, 0.02)	0.02 (0.01, 0.03)***	0.00 (−0.01, 0.01)	0.01 (0.00, 0.03)	0.00 (−0.01, 0.02)	0.00 (−0.02, 0.02)
Year^c^ × perceived overweight	0.00 (−0.01, 0.01)	0.01 (0.00, 0.02)	0.01 (0.00, 0.02)*	0.01 (0.00, 0.02)**	0.02 (0.01, 0.03)***	0.02 (0.01, 0.03)***

* *p* ≤ .05, ** *p* ≤ .01, *** *p* ≤ .001

^a^Ordinal regression (OR ±95% CI) (high values represent higher frequency of confidence or happiness)

^b^Between 1990 and 1998, participants could also select that “I don’t think about it”. For the purposes of analysis, this option is coded as ‘about right’. Those responding that they think that their body is “a bit too thin” or “much too thin” are categorised ‘perceived underweight’, and those responding that their body is “a bit too fat” or “much too fat” are categorised ‘perceived overweight’

^c^Survey year centred on 1994

^d^General linear model (*B* ± 95% CI) (higher values represent more frequent health symptoms)

No linear change over time was seen in the confidence of 11- or 13-year-old boys perceiving themselves as ‘about right’. However, the equivalent group among 15-year-old boys exhibited reducing levels of confidence between 1994 and 2014, as did 13- and 15-year-old girls without under- or overweight perception. Eleven-year-old girls perceiving their body as ‘about right’ exhibited increasing levels of confidence between 1994 and 2014. For all age/gender permutations, an interaction was observed between survey year and perceived overweight. Relative to those perceiving their body as ‘about right’, adolescents perceiving themselves as overweight have, over time, become increasingly likely to report low confidence.

#### Happiness

**Fig. 2:**
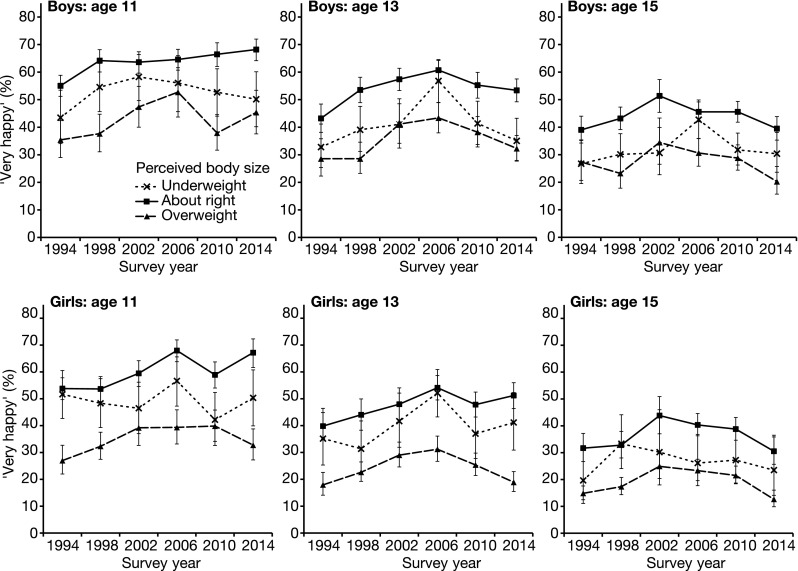
Relationship between Scottish adolescents’ happiness and perceived body size 1994–2014. The proportion of boys and girls aged 11, 13, and 15 reporting that they are “very happy” is presented for those perceiving their body is underweight (*dashed line*, *crosses*), overweight (*dashed line*, *triangles*), or ‘about right’ (*solid line*, *squares*). For the 1994 and 1998 surveys, participants could also select that “I don’t think about it”. For the purposes of analysis, this option is coded as ‘about right’. *Error bars* represent ±95% CI. Scotland 1994–2014

The relationship between happiness and perceived body size over the period 1994–2014 is demonstrated in Fig. 2. This relationship is formally examined in Table 3, which presents the results of ordinal regressions testing main effects of body size perception and survey year, and their interactive effect on happiness for each age/gender permutation. In contrast to the findings for girls, boys in all three age groups perceiving themselves as underweight exhibited reduced happiness relative to those that perceive themselves as ‘about right’. Perceived overweight was associated with reduced happiness for all age/gender permutations.

Whilst happiness increased among 11- and 13-year-old girls perceiving their body as ‘about right’ between 1994 and 2014, their counterparts perceiving themselves as overweight did not improve over this period, thus a relative reduction in levels of happiness was seen for these individuals. Eleven-year-old girls perceiving themselves as underweight also experienced a relative reduction in happiness over time.

Eleven and 13-year-old boys perceiving their body as ‘about right’ also became increasingly happy between 1994 and 2014. No interaction between survey year and perceived over- or underweight was seen among boys of any age.

#### Psychological health symptoms

**Fig. 3:**
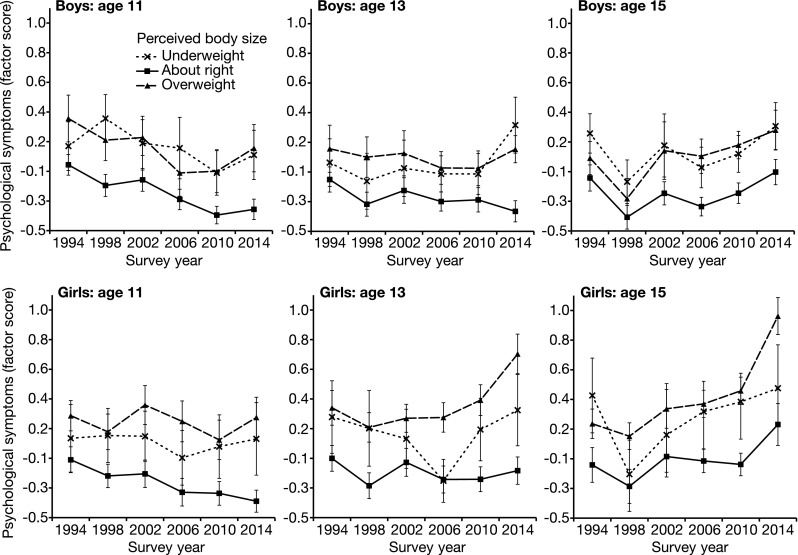
Relationship between Scottish adolescents’ psychological health symptoms and perceived body size 1994–2014. Principal components analysis was used to generate an index of the following psychological symptoms: nervousness, bad temper, feeling low, and sleep difficulties, with higher scores representing more frequent symptoms. The mean psychological symptom factor score for boys and girls aged 11, 13, and 15 is presented for those perceiving their body is underweight (*dashed line*, *crosses*), overweight (*dashed line*, *triangles*), or ‘about right’ (*solid line*, *squares*). For the 1994 and 1998 surveys, participants could also select that “I don’t think about it”. For the purposes of analysis, this option is coded as ‘about right’. *Error bars* represent ±95% CI. Scotland 1994–2014

The relationship between psychological symptoms and perceived body size over the period 1994–2014 is demonstrated in Fig. 3. This relationship is formally examined in Table 3, which presents the results of general linear models testing the main effects of body size perception and survey year, and their interactive effect on psychological symptoms for each age/gender permutation. Perceived underweight was associated with increased psychological symptoms for 11- and 15-year-old boys and girls of all ages. For all age/gender permutations, perceived overweight was associated with increased psychological symptoms.

For girls of all ages and 15-year-old boys, an interaction was observed between survey year and perceived overweight. Relative to those perceiving their body as ‘about right’, those perceiving themselves as overweight have become increasingly likely to report psychological symptoms since 1994. Thirteen-year-old boys perceiving themselves as underweight also exhibited a relative increase in psychological symptoms over this period.

### Association between body mass index and perceived body size

A possible explanation for relative deterioration of mental well-being outcomes among those with over- or underweight perception is that the actual weight of these individuals has changed over time compared to those perceiving themselves as ‘about right’. To investigate this, time trends in body mass index (BMI) were examined in post hoc analyses (see Supplementary Table S3). The mean BMI of 15-year-old boys perceiving themselves as overweight reduced between 2006 and 2014 relative to those perceiving themselves as ‘about right’ [*F* (1771) = 4.54, *p* = .033]. For all other age/gender permutations, no relative changes in mean BMI were seen between 2006 and 2014 for those with over- or underweight perceptions (all *F* < 1.64, *p* > .201).

## Discussion

Data from the Scottish HBSC study were used to examine age differences and trends in adolescents’ under- and overweight perceptions between 1990 and 2014. We also investigated whether the association between perceived body size and mental well-being has changed over the period between 1994 and 2014.

### Trends in body size perception

Among boys, there was little change in the prevalence of under- or overweight perceptions, the only substantive change being for 15-year-old boys, who, since 1990, have become more likely to report that they are overweight. Conversely, since 1990, 11-year-old girls have become less likely to report that they are either underweight or overweight. Whilst 13- and 15-year-old girls have also become less likely to perceive themselves as underweight, they have consistently remained at high risk of perceived overweight with little change in the proportion reporting that they are overweight over this period. These findings may suggest that national body image initiatives such as the UK government’s Body Confidence Campaign (established November 2010) have been most effective in widening the perception of normative body size among pre-, but not peri- or post-pubertal girls. Our results may also indicate either that post-pubertal boys are increasingly sensitive to cultural influences on body image perception or that the nature of cultural norms is changing for this age group of males.

### Association between body size perception and mental well-being outcomes

Corroborating prior research (Cash and Fleming 2002; Atlantis and Ball 2008; Williams and Currie 2000), we demonstrate that perceived overweight is strongly associated with reduced confidence and happiness, and increased psychological symptoms for boys and girls at all observed ages. Consequently, the high and largely unabating prevalence of perceived overweight among young people in Scotland is concerning, with currently over 40% of 11–15-year-old girls and a quarter of boys feeling that they are overweight. Moreover, our findings suggest that the recently observed deterioration in Scottish adolescents’ mental well-being (Currie et al. 2015) may be precipitated in part by a worsening association between mental well-being and perceived overweight, especially among girls. Whilst for some groups, we did not observe evidence of this worsening association across all mental well-being outcomes (predominantly boys; e.g., 11- and 13-year-old boys’ psychological symptoms), this tended to be among groups experiencing lesser deterioration in these outcomes over time (Currie et al. 2015).

Whilst the prevalence of perceived underweight has remained comparatively low since 1990, it too was associated with reduced mental well-being, particularly among boys. These findings are concordant with indications elsewhere (Leone et al. 2005) that muscle dysmorphia is an issue that affects adolescent boys’ mental well-being. We present some evidence that perceived underweight may impose an increasingly negative effect on adolescents’ mental well-being; however, this evidence is much less consistent than that for perceived overweight.

Perceived underweight was associated with increased psychological health symptoms for both boys (except 13-year-olds) and girls. Late pubertal development is generally held to be a factor in adolescent boys’ underweight perceptions (Siegel et al. 1999). It may also be the case that perceived underweight amongst girls has a similar aetiology, arising due to physical discrepancy between self and more physically developed peers (Petersen and Taylor 1980). Further work with cross-sectional and longitudinal data sets will elucidate links between pubertal timing, perceived body size and mental well-being, and whether any change over time in the role of pubertal timing is associated with changes in mental well-being outcomes.

For some groups, well-being outcomes have worsened over time even among those reporting normal body size (e.g., the confidence and psychological symptoms of 15-year-old boys and girls). Whilst there are likely to be determinants of declining mental well-being beyond those concerning body image, it is important to recognise that broader aspects of self-perception may also be relevant in this context. Low levels of overall appearance satisfaction exist among Scottish adolescents, even for those perceiving normal weight. In 2014, 42% of Scottish girls aged 13 and 15 reported that their body is about the right size, but only 12% reported that they have good looks (Currie et al. 2015). To resolve their relative importance, it is necessary for further work to simultaneously examine the influence of weight-dependent and weight-independent appearance concerns on mental well-being outcomes, particularly in Scotland where girls’ self-perceived attractiveness has reduced substantially since 2002 (Currie et al. 2015).

### Limitations

Given the cross-sectional design of the HBSC study, a limitation of this study is that we cannot be certain that the changing association between body weight perception and mental well-being is causative, although our results are consistent with this explanation. There may exist unexamined factors which have changed over the studied time period, (e.g., quality of peer relationships) which confound the apparent effect of time in this analysis. There also may exist factors associated both with perceived body size and mental well-being that are the true drivers of worsening well-being. One such factor is BMI (Gray and Leyland 2008; Quick et al. 2014). Our post hoc analyses suggested that the worsening association between weight perception and mental well-being is not due to those perceiving themselves as overweight experiencing a relative increase in weight. However, data on adolescents’ BMI were only available from 2006 onwards. In addition, we were restricted to self-reported height and weight, with a large amount of missing, and potentially biased data (Elgar and Stewart 2008), which could obscure any relative increase in the weight of those perceiving they are overweight; however, we cannot definitively say that this is the case given the available data. Socioeconomic status is also a potential confound which has previously been related to both body weight perception and mental well-being (Inchley et al. 2016; McLaughlin et al. 2012). Future work in this context is warranted amongst adolescents from nations with positive or negative associations between affluence and perceived overweight, such as Turkey and Iceland, respectively (Inchley et al. 2016).

### Conclusion

Whilst there has been little change since 1990 in the prevalence of overweight perceptions, our findings suggest that perceiving oneself as overweight has a larger impact on Scottish adolescents’ mental well-being today than it did 20 years ago. In addition to an increasing impact on psychological well-being, we speculate that perceived overweight may also be increasingly responsible for poor somatic health. Adolescents experiencing overweight perceptions and mental health symptoms are less likely to engage in health-promoting activities, including those important for maintaining a healthy weight (Fulkerson et al. 2004; Loth et al. 2015; Neumark-Sztainer et al. 2006; Robinson et al. 2015). It is possible, then, that the focus on weight-related issues accompanying the global obesity epidemic (Brownell et al. 2010; Taylor 2011) is increasingly responsible for exacerbating, rather than ameliorating weight-related morbidity.

When targeting obesity via public health campaigns, it is crucial to consider how to incentivize weight loss without damaging adolescents’ self-perception (O’Dea 2005). Those individuals who are overweight may be particularly likely to perceive a wide gap between current and ideal body image. Therefore, these individuals may benefit from more achievable weight-loss goals in conjunction with techniques to enhance self-efficacy (Linde et al. 2006) and psychological resilience. Future observation of national and international trends in body perceptions will help evaluate whether contemporary body image campaigns (e.g., the ‘Be Real’ campaign) are effective in both widening the definition of normative body weight and in encouraging positive body image perception among young people, particularly non-overweight individuals considering themselves as overweight.

Adolescence presents particular challenges as a time of marked physical development and identity formation. Efforts are required to help support young people through this transition period to protect and enhance self-perceptions and well-being. It will be valuable to explore potential mediators and moderators of the changing relationship between body image and mental well-being outcomes, such as digital media use, physical activity, bullying, and socio-cultural assets (Fenton et al. 2010) to shape future intervention efforts.

## Electronic supplementary material

Below is the link to the electronic supplementary material.

Supplementary material 1 (PDF 195 kb)

## References

[CR1] Atlantis E, Ball K (2008). Association between weight perception and psychological distress. Int J Obes.

[CR2] Blashill AJ, Wilhelm S (2014). Body image distortions, weight and depression in adolescent boys: longitudinal trajectories into adulthood. Psychol Men Masc.

[CR3] Blowers LC, Loxton NJ, Grady-Flesser M, Occhipinti S, Dawe S (2003). The relationship between sociocultural pressure to be thin and body dissatisfaction in preadolescent girls. Eat Behav.

[CR4] Brownell KD, Kersh R, Ludwig DS (2010). Personal responsibility and obesity: a constructive approach to a controversial issue. Health Aff.

[CR5] Cash TF, Fleming EC, Cash TF, Pruzinsky T (2002). Body image and social relations. Body image: a handbook of theory, research, and clinical practice.

[CR6] Cash TF, Szymanski ML (1995). The development and validation of the body-image ideals questionnaire. J Pers Assess.

[CR7] Currie C, Levin K, Kirby J, van der Sluijs W, Inchley J (2011) Health Behaviour in School-aged Children: World Health Organization Collaborative Cross-national Study (HBSC): findings from the 2010 HBSC survey in Scotland. Child and Adolescent Health Research Unit http://www.cahru.org/downloads/HBSC_National_Report_2010_LowRes.pdf. Accessed 06 June 2016

[CR8] Currie C, Inchley J, Molcho M, Lenzi M, Veselzka Z, Wild F (2014) Health Behaviour in School-aged Children (HBSC). Study protocol: background, methodology and mandatory items for the 2013/2014 survey. Child and Adolescent Health Research Unit. https://docs.google.com/a/hbsc.org/forms/d/19lHQZls37M2xeEDr_K2TxWba39HS7h4UPXJZuA_9k4c/viewform?formkey=dEFYVURnWHctUVhvM0gxRlVkVTVLOXc6MQ#gid=0. Accessed 06 June 2016

[CR9] Currie C, van der Sluijs W, Whitehead R et al (2015) Health Behaviour in School-aged Children: World Health Organization Collaborative Cross-national Study (HBSC): findings from the 2014 HBSC survey in Scotland. Child and Adolescent Health Research Unit. http://www.cahru.org/content/03-publications/04-reports/hbsc_nr14_interactive_final.pdf. Accessed 06 June 2016

[CR10] Elgar FJ, Stewart JM (2008). Validity of self-report screening for overweight and obesity. Evidence from the Canadian Community Health Survey. Can J Public Health.

[CR11] Fenton C, Brooks F, Spencer NH, Morgan A (2010). Sustaining a positive body image in adolescence: an assets-based analysis. Health Soc Care Community.

[CR12] Friestad C, Rise J (2004). A longitudinal study of the relationship between body image, self-esteem and dieting among 15–21 year olds in Norway. Eur Eat Disord Rev.

[CR13] Fulkerson JA, Sherwood NE, Perry CL, Neumark-Sztainer D, Story M (2004). Depressive symptoms and adolescent eating and health behaviors: a multifaceted view in a population-based sample. Prev Med.

[CR14] Gray L, Leyland AH (2008). Overweight status and psychological well-being in adolescent boys and girls: a multilevel analysis. Eur J Public Health.

[CR15] Haugland S, Wold B, Stevenson J, Aaroe LE, Woynarowska B (2001). Subjective health complaints in adolescence. A cross-national comparison of prevalence and dimensionality. Eur J Public Health.

[CR16] Inchley J, Currie D, Young T et al (2016) Growing up unequal: gender and socioeconomic differences in young people’s and well-being. Health Behaviour in School-aged Children (HBSC) study: international report from the 2013/2014 survey. WHO Regional Office for Europe. http://www.euro.who.int/en/health-topics/Life-stages/child-and-adolescent-health/health-behaviour-in-school-aged-children-hbsc. Accessed 06 June 2016

[CR17] Leone JE, Sedory EJ, Gray KA (2005). Recognition and treatment of muscle dysmorphia and related body image disorders. J Athl Train.

[CR18] Linde JA, Rothman AJ, Baldwin AS, Jeffery RW (2006). The impact of self-efficacy on behavior change and weight change among overweight participants in a weight loss trial. Health Psychol.

[CR19] Loth KA, Watts AW, van den Berg P, Neumark-Sztainer D (2015). Does body satisfaction help or harm overweight teens? A 10-year longitudinal study of the relationship between body satisfaction and body mass index. J Adolesc Health.

[CR20] McLaughlin KA, Costello EJ, Leblanc W, Sampson NA, Kessler RC (2012). Socioeconomic status and adolescent mental disorders. Am J Public Health.

[CR21] Neumark-Sztainer D, Paxton SJ, Hannan PJ, Haines J, Story M (2006). Does body satisfaction matter? Five-year longitudinal associations between body satisfaction and health behaviors in adolescent females and males. J Adolesc Health.

[CR22] O’Dea JA (2005). Prevention of child obesity: ‘First, do no harm’. Health Educ Res.

[CR23] Petersen AC, Taylor B, Adelson J (1980). The biological approach to adolescence: biological change and psychological adaptation. Handbook of adolescent psychology.

[CR24] Quick V, Nansel TR, Liu D, Lipsky LM, Due P, Iannotti RJ (2014). Body size perception and weight control in youth: 9-year international trends from 24 countries. Int J Obes.

[CR25] Robinson E, Hunger JM, Daly M (2015). Perceived weight status and risk of weight gain across life in US and UK adults. Int J Obes.

[CR26] Siegel JM, Yancey AK, Aneshensel CS, Schuler R (1999). Body image, perceived pubertal timing, and adolescent mental health. J Adolesc Health.

[CR27] Strahan EJ, Wilson AE, Cressman KE, Buote VM (2006). Comparing to perfection: how cultural norms for appearance affect social comparisons and self-image. Body Image.

[CR28] Sypeck MF, Gray JJ, Ahrens AH (2004). No longer just a pretty face: fashion magazines’ depictions of ideal female beauty from 1959 to 1999. Int J Eat Disord.

[CR29] Taylor NL (2011). Negotiating popular obesity discourses in adolescence school food, personal responsibility, and gendered food consumption behaviors. Food Cult Soc.

[CR30] Voelker DK, Reel JJ, Greenleaf C (2015). Weight status and body image perceptions in adolescents: current perspectives. Adolesc Health Med Ther.

[CR31] Williams JM, Currie C (2000). Self-esteem and physical development in early adolescence: pubertal timing and body image. J Early Adolesc.

[CR32] World Health Organization (2015) Obesity and overweight. Factsheet N 311. http://www.who.int/mediacentre/factsheets/fs311/en/. Accessed 06 June 2016

