# Supplementary material for: Trends in the perceived body size of adolescent males and females in Scotland, 1990–2014: changing associations with mental well-being
Source: Int J Public Health. 2017 Jul 1;63(1):69–80. doi: 10.1007/s00038-017-0997-y (PMC5766710; doi:10.1007/s00038-017-0997-y)
Supplement: Supplementary file 1 — Supplementary material 1 (PDF 195 kb) [file 38_2017_997_MOESM1_ESM.pdf]

Trends in the Perceived Body Size of Adolescent Males and Females in Scotland, 1990-2014: Changing Associations with Mental Well-being

International Journal of Public Health

Ross Whitehead Ph.D. [1], Alina Cosma Ph.D. [1], Jo Cecil Ph.D. [1], Candace Currie Ph.D. [1], Dorothy Currie MSc. [1], Fergus Neville Ph.D. [1] and Jo Inchley Ph.D. [1]
[1] School of Medicine, University of St Andrews, St Andrews, Fife, Scotland.

Corresponding author: rw394@st-andrews.ac.uk

Table S1. Age differences and time trends in adolescents' underweight perceptions, Scotland 1990-2014 [a, b]

Boys		Girls	
**Model 1a**			
11-year olds (ref)		11-year olds (ref)	
13-year olds	1.18 (1.07, 1.31)**	13-year olds	0.95 (0.84, 1.06)
15-year olds	1.60 (1.44, 1.77)***	15-year olds	0.72 (0.64, 0.81)***
**Model 1b**			
11-year olds	0.63 (0.56, 0.69)***	11-year olds	1.39 (1.24, 1.57)***
13-year olds	0.74 (0.67, 0.81)***	13-year olds	1.32 (1.17, 1.49)***
15-year olds (ref)		15-year olds (ref)	
**Model 2**			
Year [c]	1.00 (0.99, 1.00)	Year [c]	0.99 (0.98, 1.00)**
**Model 3a**			
11-year olds (ref)		11-year olds (ref)	
13-year olds	1.32 (1.09, 1.60)**	13-year olds	0.95 (0.78, 1.16)
15-year olds	1.87 (1.55, 2.26)***	15-year olds	0.69 (0.57, 0.85)***
Year [c]	1.00 (0.99, 1.01)	Year [c]	0.99 (0.98, 1.00)*
Year × 11-year olds (ref)		Year × 11-year olds (ref)	
Year × 13-year olds	0.99 (0.98, 1.00)	Year × 13-year olds	1.00 (0.99, 1.01)
Year × 15-year olds	0.99 (0.98, 1.00)	Year × 15-year olds	1.00 (0.99, 1.02)
**Model 3b**			
11-year olds	0.53 (0.44, 0.64)***	11-year olds	1.44 (1.18, 1.76)***
13-year olds	0.71 (0.59, 0.85)***	13-year olds	1.37 (1.09, 1.71)**
15-year olds (ref)		15-year olds (ref)	
Year [c]	0.99 (0.98, 1.00)	Year [c]	0.99 (0.98, 1.00)
Year [c] × 11-year olds	1.01 (1.00, 1.02)	Year [c] × 11-year olds	1.00 (0.98, 1.01)
Year [c] × 13-year olds	1.00 (0.99, 1.02)	Year [c] × 13-year olds	1.00 (0.98, 1.01)
Year [c] × 15-year olds (ref)		Year [c] × 15-year olds (ref)	

[a] Logistic regression (OR ± 95% CI). [b] Those responding that they think their body is "a bit too thin" or "much too thin" are categorised 'perceived underweight', with all other options "a bit too fat", "much too fat", "about right", and "I don't think about it" (the latter only available between 1990 and 1998) categorised as 'not perceived underweight'. [c] Survey year centred on 1990. * p≤.05, **p≤.01, ***p≤.001

Table S2. Age differences and time trends in adolescents' overweight perceptions, Scotland 1990-2014 [a, b]

Boys		Girls	
**Model 1a**			
11-year olds (ref)		11-year olds (ref)	
13-year olds	1.45 (1.33, 1.58)***	13-year olds	1.95 (1.78, 2.12)***
15-year olds	1.33 (1.21, 1.45)***	15-year olds	2.72 (2.50, 2.96)***
**Model 1b**			
11-year olds	0.75 (0.69, 0.83)***	11-year olds	0.37 (0.34, 0.40)***
13-year olds	1.09 (1.01, 1.19)*	13-year olds	0.72 (0.66, 0.77)***
15-year olds (ref)		15-year olds (ref)	
**Model 2**			
Year [c]	1.01 (1.00, 1.01)**	Year [c]	0.99 (0.99, 1.00)*
**Model 3a**			
11-year olds (ref)		11-year olds (ref)	
13-year olds	1.28 (1.09, 1.51)**	13-year olds	1.37 (1.18, 1.59)***
15-year olds	0.97 (0.82, 1.16)	15-year olds	2.04 (1.77, 2.35)***
Year [c]	1.00 (0.99, 1.01)	Year [c]	0.98 (0.97, 0.98)***
Year × 11-year olds (ref)		Year × 11-year olds (ref)	
Year × 13-year olds	1.01 (1.00, 1.02)	Year × 13-year olds	1.03 (1.02, 1.04)***
Year × 15-year olds	1.02 (1.01, 1.03)***	Year × 15-year olds	1.02 (1.01, 1.03)***
**Model 3b**			
11-year olds	1.03 (0.86, 1.22)	11-year olds	0.49 (0.43, 0.57)***
13-year olds	1.32 (1.11, 1.56)**	13-year olds	0.67 (0.58, 0.78)***
15-year olds (ref)		15-year olds (ref)	
Year [c]	1.02 (1.01, 1.03)***	Year [c]	1.00 (0.99, 1.01)
Year [c] × 11-year olds	0.98 (0.97, 0.99)***	Year [c] × 11-year olds	0.98 (0.97, 0.99)***
Year [c] × 13-year olds	0.99 (0.98, 1.00)*	Year [c] × 13-year olds	1.00 (0.99, 1.01)
Year [c] × 15-year olds (ref)		Year [c] × 15-year olds (ref)	

[a] Logistic regression (OR ± 95% CI). [b] Those responding that they think their body is "a bit too fat" or "much too fat" are categorised 'perceived overweight', with all other options "a bit too thin", "much too thin", "about right", and "I don't think about it" (the latter only available between 1990 and 1998) categorised as 'not perceived overweight'. [c] Survey year centred on 1990. * $p \leq .05$, ** $p \leq .01$, *** $p \leq .001$

Table S3. Associations between perceived body size and body mass index, Scotland 2006-2014 [a]

	Boys			Girls		
	**11-year olds**	**13-year olds**	**15-year olds**	**11-year olds**	**13-year olds**	**15-year olds**
'About right' [b] (ref)						
Perceived underweight	-2.10 (-2.78, -1.41)***	-1.96 (-2.59, -1.32)***	-1.89 (-2.29, -1.49)***	-2.03 (-2.70, -1.37)***	-2.07 (-2.62, -1.51)***	-2.17 (-2.75, -1.58)***
Perceived overweight	3.41 (2.49, 4.33)***	3.08 (2.42, 3.74)***	3.56 (2.90, 4.22)***	3.02 (2.23, 3.82)***	2.92 (2.24, 3.60)***	2.61 (2.13, 3.10)***
Year [c]	-0.02 (-0.10, 0.06)	0.10 (0.03, 0.16)**	0.07 (0.01, 0.13)*	-0.05 (-0.16, 0.06)	-0.01 (-0.09, 0.08)	0.06 (-0.01, 0.14)
Year [c] × 'About right' [b] (ref)						
Year [c] × Perceived underweight	-0.01 (-0.14, 0.13)	-0.06 (-0.20, 0.07)	0.01 (-0.10, 0.12)	-0.05 (-0.21, 0.11)	0.04 (-0.07, 0.16)	-0.07 (-0.22, 0.08)
Year [c] × Perceived overweight	-0.08 (-0.28, 0.12)	-0.03 (-0.19, 0.14)	-0.16 (-0.30, -0.01)*	0.01 (-0.16, 0.18)	-0.09 (-0.26, 0.08)	-0.08 (-0.19, 0.04)

[a] General linear model (*B* ± 95% CI). [b] Those responding that they think their body is "a bit too thin" or "much too thin" are categorised 'perceived underweight', and those responding that their body is "a bit too fat" or "much too fat" are categorised 'perceived overweight'. [c] Survey year centred on 2006. * $p \leq .05$, ** $p \leq .01$, *** $p \leq .001$
